# An Overview of Murine High Fat Diet as a Model for Type 2 Diabetes Mellitus

**DOI:** 10.1155/2016/2902351

**Published:** 2016-07-31

**Authors:** Ahlke Heydemann

**Affiliations:** ^1^The University of Illinois at Chicago, Chicago, IL 60612, USA; ^2^The Center for Cardiovascular Research, Chicago, IL 60612, USA

## Abstract

Type 2 diabetes mellitus (T2DM) is a worldwide epidemic, which by all predictions will only increase. To help in combating the devastating array of phenotypes associated with T2DM a highly reproducible and human disease-similar mouse model is required for researchers. The current options are genetic manipulations to cause T2DM symptoms or diet induced obesity and T2DM symptoms. These methods to model human T2DM have their benefits and their detractions. As far as modeling the majority of T2DM cases, HFD establishes the proper etiological, pathological, and treatment options. A limitation of HFD is that it requires months of feeding to achieve the full spectrum of T2DM symptoms and no standard protocol has been established. This paper will attempt to rectify the last limitation and argue for a standard group of HFD protocols and standard analysis procedures.

## 1. Introduction

There are a number of open issues in the HFD mouse modeling of T2DM:

- Will these mouse models be useful in preclinical investigations?
- What about the differences identified between wild type mouse strains?
- Why some protocols do not elicit DCM?
- Can the research community agree upon a common HFD feeding protocol? Comparisons between papers, genetic manipulations, and therapies will be much easier and more informative if a specific diet, length of diet, and analysis protocols can be agreed upon.

An updated murine HFD review is urgently required as 223 references for January 2016 were found on PubMed using “high fat diet and mouse” as the search term (too many to read all). The presentation of such a large and highly important body of work is daunting. The body of this review will therefore be focused upon a researcher's point of view. The goal is that researchers will use this paper to plan their HFD protocols and to create some consistency in future experimental protocols and analysis techniques.

Due to differences in the literature, there are* multiple terms* that should be defined before moving forward. We will use the following convention:* obesity* leads to* metabolic syndrome* which can progress to* T2DM* [1]. In humans obesity is defined by a body mass index (BMI = weight/height^2^) of over 30 kg/m^2^. Progression to metabolic syndrome is diagnosed when a patient has 3 of the following 5 pathologies: obesity, hypertension, fasting hyperglycemia, elevated serum triglycerides, and decreased high-density lipoprotein. Metabolic syndrome is often labeled as prediabetes. T2DM is initially defined as a patient who is insulin resistant. After years of the associated hyperinsulinemia the pancreas may falter and the patient will then also suffer from hypoinsulinemia. Type 1 diabetes is a disease of the pancreas itself. The pancreas fails to produce insulin and therefore the patient becomes hyperglycemic, but never hyperinsulinemic.

*Mouse models have proven invaluable* in acquiring basic knowledge about human diseases. This knowledge progresses to preclinical investigations in these same mouse models. Examples of knowledge and therapies which have progressed from basic knowledge to preclinical trials in mouse models are numerous ([2] and some examples are reviewed in [3–5]). In the obesity and T2DM fields, mouse models have proven invaluable in the basic science of the diseases by identifying the roles of inflammation, insulin resistance, fat content of the diet, pAMPK, exercise, and potential treatments. In addition, and very importantly, what has been learned from the mouse models has faithfully been carried over into the human patients [6]. These physiological similarities between the two species are due to the genetic homology between the two species [7].

One of the largest problems—or perhaps one of the most advantageous aspects—of mouse models for T2DM are the* different pathologic responses between mouse strains.* These differences are also apparent in many other murine models of human disease. Multiple publications have identified strain specific differences in HFD susceptibility (a few recent examples are [8–13]). The advantageous aspects of these differences lie in the pursuit of mechanisms which cause a mouse strain to be resistant to HFD pathologies and therefore provide avenues to identify targets for future therapies. However, researchers must also be aware of these phenotypic differences when making comparisons with earlier publications which have utilized different mouse strains or even different sources for a mouse strain [13].

In the HFD field there are also* disparities regarding the development of diabetic cardiomyopathy* (DCM). Many manuscripts have identified DCM after a relatively short diet intervention [14–17] while other experiments did not identify DCM after an 8-month HFD protocol [18] or 6-month HFD protocol [19]. Some of these disparities are easily identified to lie in specific HFD formulations or specific mouse strains being utilized or even to know which characteristics of DCM are being considered for comparison. However, some of the disparities are not so easily identified and having standardized HFD protocols will aid in identifying where the differences are arising and if they are worth pursuing for increased T2DM mechanistic knowledge. For further insights into DCM the reader is revered to a recent review article [5] concerning DCM in both T1DM and T2DM mouse models.

Of further interest to researchers is that* mice subjected to HFD dependably model other human conditions* in addition to T2DM. Among these other diseases are those usually related to T2DM but not occurring in all patients or mice. Nonalcoholic fatty liver disease (NAFLD) and the progression of this disease to nonalcoholic steatohepatitis (NASH) and hepatocellular carcinoma (HCC) are common sequela of obesity and T2DM [20]. These conditions are well modeled with murine long-term HFD strategies (reviewed in [21]). HFD has also been utilized to model chronic inflammation, which is an important pathogenic mechanism of T2DM [22] and many other diseases including aging. The HFD-mediated chronic inflammation is marked by increased TNF-*α*, IL-1*β*, and IL-6 in the circulation [23]. Among the additional inflammatory diseases investigated using murine HFD are wound healing [24, 25], prostate disease [26], dysbiosis [27–29], aging [30], and Alzheimer's [31]. The HFD mice also provide very good models for investigating exercise benefits [32] and muscle regeneration [33]. Researchers have identified that murine HFD successfully models key aspects of peripheral artery disease [34]. HFD has also been shown to decrease skeletal muscle regeneration, likely through inflammation and lipotoxicity in the muscle stem cells known as satellite cells (reviewed in [35]). Furthermore, impaired leptin signaling which occurs in HFD fed mice also decreases satellite cell proliferation [36].

The* evolutionary aspects of T2DM and insulin resistance* are also highly interesting. Insulin resistance occurs in many tissues, of primary importance in the skeletal muscle, liver, and heart. Using acute insulin challenge protocols described below, individual, specific tissues can be analyzed for their level of insulin resistance. The “thrifty genome” theory posits that slight insulin resistance may have had some advantage when our ancestors (and the ancestors of mice in the wild) underwent times of food scarcity [37]. This transient insulin resistant after a large meal would cause increased calorie storage for the following times of food scarcity. However, now during times of constant food overabundance in many cultures, and for the mice, the transient and slight insulin resistance has become chronic and leads to pathologies. Although recent reports do not support the theory [38–40], there must be an evolutionary reason for the maintenance of loci associated with T2DM. An additional theory presents that insulin resistance can protect the heart from excessive calories [41]. This is a very important idea when considering insulin sensitizing agents as therapy, which, if this theory is valid, would cause further cardiac damage as has been seen with rosiglitazone treatment [41, 42]. In either case, HFD in mice has been a highly effective tool in investigating the phenotypic mechanisms and therapeutic possibilities.

A number of* recent publications and reviews* have addressed the utility of the mouse model HFD protocol. These reviews have excellent details and I will therefore refer the reader to these details at appropriate sections, instead of being repetitive. Calligaris et al. discuss the cardiac disease phenotype of murine HFD feeding and present an important time course of phenotypic progression [18]. A recent review article presents the use of experimental models for diabetic cardiomyopathy [5]. Islam and Loots very competently present the similarities between the murine HFD model and humans suffering from T2DM [6]. Another review manuscript describes the use of HFD mice to identify factors involved in obesity resistance or sensitivity [43]. A recent review has described various metabolic phenotyping methodologies in detail [44]. In addition, a recent chapter has described the utility of mouse models for T2DM drug discovery [4]. In addition, a thorough discussion of insulin resistance in multiple tissues was recently published [45]. Therefore, these topics will not be presented here in detail.

Additional publications describe* genetically engineered T2DM mouse models,* which have recently been reviewed [46]. However, due to the many genes identified which cause T2DM—more than 50 genes having been identified by human GWAS studies [47]-none of these murine genetic models can model disease etiology of more than a few patients. Many of these mice faithfully recapitulate some of the T2DM phenotypes and can therefore be useful for preclinical trials and can be used to investigate specific portions of the phenotype. However, they do not model the disease etiology of the majority of patients [46]. Most T2DM humans have become sick due to their diets, not their genetics (CDC, http://www.cdc.gov/diabetes/data/statistics/faqs.html). Therefore the HFD is the most appropriate disease model [18].

## 2. Considerations for Designing a High Fat Diet Protocol

There are many important considerations for designing a murine HFD protocol (Table 1). The most important is perhaps the* duration of the HFD.* Other considerations are the* age of the mice* and* formulation of the HFD.* As discussed, which* mouse strain* to use is also highly important to consider when designing a HFD protocol.

We investigated many manuscripts and identified many* different durations* for diet intervention; the most commonly utilized times are listed in Table 2. Clearly this wide array of times (and diet specifics and age at start) makes interstudy comparisons very difficult. Early during diet intervention (<1 week) we expect an adaptation phase. Such an early adaptation phase was noted in the HFD fed wild type MRL mouse strain after 2 and 3 weeks of HFD [12]. The MRL mice became slightly hyperglycemic at these early weeks, which they fully recovered from by 7 weeks of diet. After a certain HFD length we expect some parameters to have achieved a plateau. Such plateaus are evident for weekly hyperglycemia values and weight gain [12]. However some very important disease parameters will continue to worsen. Examples of continuing pathogenic characteristics are insulin resistance [60], inflammation [48], and cardiac remodeling [18].

The earliest HFD effects we found in the literature were after 3 days of diet as an increase of pancreatic *β*-cell proliferation [60]. These authors also followed a time course through 11 weeks of HFD which revealed important differences between early (1 week) and late (11 weeks) results in intraperitoneal GTT and the dynamics of disease progression. Insulin tolerance also progressively worsened over the time course, while hyperinsulinemia was only apparent at 11 weeks of diet and was not seen at 1 or 5 weeks of diet [60]. This is a great illustration that the length of time of HFD must be carefully considered depending upon which variables one is interested in investigating. Furthermore, the above-mentioned early adaptation phase seen in the MRL mice did not occur in the HFD fed C57Bl/6 mice, again stressing the importance of choosing the correct mouse strain depending upon the desired investigations.

Continuing data is being generated indicating* gender differences* in response to HFD protocols. For example, male mice are more susceptible to hyperglycemia from HFD [61]. Others have identified adipocyte baseline differences that is in wild type mice fed a normal diet between male and female mice in glucose metabolism [62]. In a highly comprehensive study Morselli et al. identified that due to HFD changes in the C57Bl/6 male hypothalamus the male mice were more susceptible to HFD-mediated chronic inflammation, while the female mice gained as much weight; they did not suffer from the pathogenic inflammation [63]. Similarly, it has been identified that multiple fat deposits in C57Bl/6T female mice and castrated male mice are more insulin sensitive than those from intact male mice [62]. These differences must be taken advantage of to find novel therapeutic targets.

It is well known that* age-related* chronic inflammation correlates strongly with insulin resistance [64]. As T2DM is still considered a disease of the elderly it is fully appropriate to analyze older mice. However, unless one is contrasting age, comparisons with younger mice must be made with caution.

A great example of the variable responses of* different mouse strains* to a HFD was recently published [48]. The authors compared the C57Bl/6 mouse strain, containing a typical Th1 leaning immune system, to the BALB/c mouse strain which is a Th2 leaning strain. Liver phenotypes and immune responses were assessed. The authors identified that the C57Bl/6 mice were more susceptible to adiposity, liver inflammation, and liver fibrosis. Alternatively, the BALB/c mice were more susceptible to liver steatosis. Furthermore, slight genetic differences due to genetic drift result in significant differences between mouse substrains [13, 65, 66]. These authors compared C57Bl/6J to C57Bl/6N substrains [13]. These strains were physically separated in 1951 and kept in different facilities since then. As no selection pressure was applied the current genetic differences are purely due to genetic drift. The C57Bl/6J strain has a mutation in nicotinamide nucleotide transhydrogenase [67]. The strains are different in the severity of HFD-induced characteristics; the C57Bl/6N strain has a milder phenotype [13]. Another advanced genetic analysis was conducted on some of the BXD mouse strains (C57BL/6J and DBA/2J intercrosses for 20 plus generations,). These authors identified strains that were susceptible and resistant to hippocampal dysfunction elicited by HFD [49]. The advantage of this strategy is that genome wide association studies and additional genetic analyses will be very informative due to the complex but known sequences [68].

A further caution must be mentioned for the variations in* diet compositions* particularly important in light of continued data that the type of the fat is critically important to provide protection or pathology [69]. The complete review of diets is beyond the scope of this review; here are a few important points to consider. In the United States there are a few main suppliers of main suppliers of lab diets: Teklad (http://Envigo.com/), LabDiet/Purina (http://labdiet.com/), Research Diets (http://researchdiets.com/), and Bio-Serve (http://bio-serve.com/). Teklad has two main HFD options with 2 modifications for one of them. In addition, Teklad provides many fat additives to custom make diets with specific lipid types. We could find two HFD in the LabDiet web pages. Research Diets sells 8 different HFD formulations. Bio-Serve sells one HFD formulation. Not only are the amount and type of fat important but also the grams of carbohydrate in each formulation are highly important to consider. As mentioned before a true “Western Diet” would have high fat and high carbohydrate.

Diets high in saturated fatty acids are more obesogenic than mono- and polyunsaturated as the saturated fatty acids are inefficiently used for energy production and are therefore more readily stored [70]. Furthermore, long-chain fatty acids (C14:0–C24:0) are more obesogenic than short- and medium-chain because they are transported into the mitochondria less readily and are therefore more likely to be stored [70]. A very interesting study regarding diet composition came from the laboratory of Ezaki. These scientists balanced carbohydrate with different lipid percentages in an isocaloric diet and demonstrated a clear correlation between lipid content and hyperglycemia [71]. As calories from lipid increased and carbohydrate calories decreased to keep total calories constant, the glucose tolerance of the mice deteriorated. Similarly, Maiolo et al. recently published comparisons of a HFD versus high sugar diet versus a diet of high fat and high sugar [72]. The results demonstrate that the combined high fat and sugar diet presented with most severe symptoms, including hyperglycemia, hypercholesterolemia, and higher levels of inflammatory mediators and lower levels of regulatory T cells. As we are all trying to improve patient lives the diet that best approximates the true Western Diet (high fat and high sugar) is arguably the best diet to use, unless diet specifics are being investigated.

Many researchers use a straightforward HFD in their protocols, but some utilize* additional stressors* to model human T2DM. An additional stressor is a low dose streptozotocin injection to boost the disease severity and model a later more progressed stage of disease after hyperinsulinemia when hypoinsulinemia has appeared [54, 73, 74]. The dose of streptozotocin does not cause any phenotypes in chow fed mice and does not always cause DCM in HFD mice. Additionally, to compensate for the additional calories provided by the HFD, some scientists have calorie that matched the amount of HFD given to their mouse cohort [75, 76]. In this way the HFD mice are only allowed to consume the same amount of calories as the CD cohort consumed in the previous 24 hours. These variations provide very useful protocols to investigate different and specific aspects of T2DM.

Although there has been speculation that sucrose with or without fat should also cause T2DM, this has not been found. In humans there is retrospective epidemiological evidence that sucrose is not causative to T2DM [77]. In mice, where the experiment can be conducted with all of the proper controls, sucrose did not alter the progression to T2DM with or without a HFD component [50]. Therefore a straightforward HFD may be the optimum diet to model early T2DM in mice with an additional streptozotocin injection to model the later stages of disease when hypoinsulinemia is also present.

## 3. Pathogenic Mechanisms

**Figure 1:**
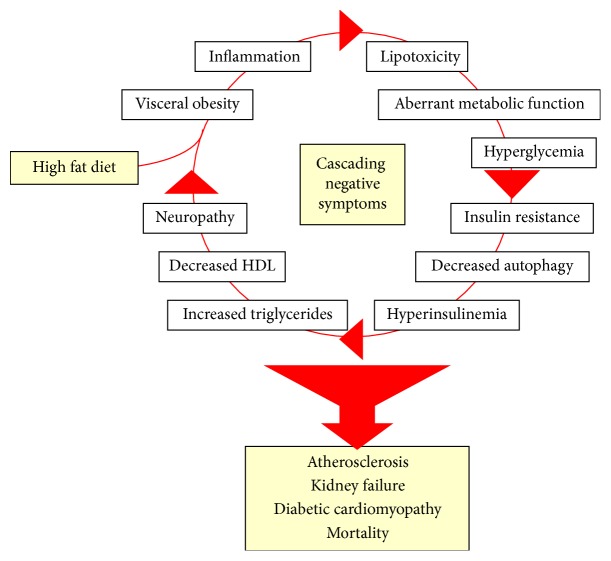
An illustration of the positive feedback nature of type 2 diabetes mellitus and murine high fat diet. Both humans and mice continue around this circle at an increasing level of pathology until they acquire one or more of the irreversible disease outcomes at the bottom of the illustration.

Type 2 diabetes mellitus (T2DM) and the murine HFD model are complex diseases with complex and overlapping pathogenic mechanisms (Figure 1). The overlapping nature of these mechanisms clearly causes researchers and clinicians anxiety when thinking about prevention and treatment strategies. For example, many patients with normoglycemia still develop downstream pathologies. Alternatively, some patients with chronically poor glucose control do not develop downstream pathologies. It is therefore clear that once T2DM is present, additional therapeutics beyond glucose control are required. Targets for the additional therapeutics require the consideration of many T2DM and HFD pathogenic mechanisms.

The direct effects of hyperglycemia upon tissues are still being fully elucidated. It appears that endothelial cells are highly sensitive to chronic hyperglycemia most possibly due to their constant proximity to the circulation. Generally it may be that the body's fuel storage cells (primarily adipose and liver cells) adapt to hyperglycemia through decreasing levels of insulin sensitivity. It may be that what is viewed as the most pathogenic portion of the disease is actually a beneficial adaptation to hyperglycemia [41]. This view is supported through evolutionary history during which humans were subjected to periods of feast or famine and during times of feast the extra calories needed to be stored in glycogen and fat deposits not utilized immediately for ATP production [78]. By temporarily shifting the insulin receptors to resistance the storage pathway would be enhanced. However, now we (humans and mice on a chronic HFD) no longer have the famine times and the resulting chronic hyperglycemia is toxic at many levels.

### 3.1. Lipotoxicity

At the onset of a HFD the murine adipose tissue adequately expands and stores the excess calories [56]. However, during chronic HFD the adipose tissue can no longer store the excess calories and the excess lipids are deposited into other organs. This can lead to disruption of normal cellular processes and then also to frank lipotoxicity where the excessive lipid molecules damage cellular molecules. Lipotoxicity occurs in multiple tissues post-HFD protocols: heart, skeletal muscle, liver pancreas, and kidneys. The definition of this pathology can be considered an imbalance in lipid uptake and disposal leading to an accumulation of lipid intermediates in nonadipose cells, which causes impaired cellular function. Excessive lipids appear to cause stress responses often leading to apoptosis and replacement of functioning cells with fibroblasts and extracellular matrix.

### 3.2. Metabolic Inflexibility

It is currently thought that healthy individuals with a varied diet and adequate exercise can adapt to their caloric source, such that eating a few HFD meals in a row will not produce any pathology. However, after chronic HFD mice and individuals become metabolically inflexible [79]. For example, cardiac tissue often responds to HFD by increasing its carbohydrate utilizing proteins to extract as much energy from glucose as possible [16, 18]. However when the diet then switches to a normal diet the heart draws too many calories from glucose causing a pathologic excessive caloric intake [41].

### 3.3. Inflammation

Inflammation is a central pathogenic mediator in all aspects of T2DM and murine HFD-induced pathologies including the cardiovascular system [80–82]. Low level chronic inflammation is identified in both T2DM and HFD mice [83]. Immune response time courses are also required as it has been shown that continued immune responses are pathogenic in many disease models, including T2DM [84]. As inflammation is also associated with many age-related diseases and conditions it becomes doubly critical in the older population historically affected by T2DM.

Multiple factors within the immune system are implicated in T2DM pathogenesis [22]. Jovicic et al. demonstrated that mice with a Th2 leaning immune response (BALB/c) are spared many of the HFD-induced pathologies, while mice leaning towards a Th1 immune response are susceptible to many HFD pathologies [48]. Infiltrating macrophages are known to be involved in pathogenesis [83].

### 3.4. Excessive Reactive Oxygen Species

A major contributor to HFD-induced DCM is hyperglycemia-induced oxidative stress [85, 86]. Hyperglycemia directly causes ROS production and apoptosis in H9c2 cells [87]. Furthermore, the mitochondrial dysfunction could be partially restored with a pharmaceutically applied antioxidant [88].

### 3.5. Decreased Autophagy

Extensive recent publications describe decreased autophagy [89, 90] and in particular mitophagy [91] as pathogenic in T2DM and HFD mouse models. Increasing autophagy can alleviate many of the HFD-mediated pathologies [92, 93]. This is clearly an ongoing and interesting research area in the HFD mice.

### 3.6. Altered Mitochondrial Morphology and Function due to Decreased Insulin Signaling

In diabetic humans hyperglycemia is thought to cause mitochondrial dysfunction and fragmentation, thereby linking hyperglycemia, mitochondrial dysfunction, and temporary insulin resistance characteristics in cardiomyocytes before obvious pathology [94]. There is significant new data that the insulin signaling cascade directly impacts cardiac mitochondrial fission and fusion (reviewed in [95]). This data arises from a number of observations in knock-out mouse models and cultured cardiomyocytes in which disrupted mitochondrial morphology and function precede T2DM [96]. The beneficial effects of metformin on increasing mitochondrial function and volume [97] are also consistent with mitochondrial pathology being pathogenic for T2DM.

## 4. Pathology Assessments

Common human T2DM and mouse HFD pathologies are as follows:

- Weight gain.
- Hyperglycemia.
- Hyperinsulinemia followed by hypoinsulinemia.
- Insulin resistance.
- Increased fasting leptin levels.
- Decreased fasting adiponectin levels.
- Inflammation with increases in inflammatory cytokines and reactive oxygen species.
- Fatty lipid droplet accumulation in multiple tissues.
- Fibrosis in multiple tissues.
- Increases in blood pressure, but not always seen in the mouse models.

There are many assays to quantify the extent of these pathologies in the HFD mouse model (Table 1). All of these assays have been described in detail, so I will just highlight some of the important considerations that may be overlooked by busy investigators and direct you to the pertinent references. Based upon my own limited experience many of these assays should be done weekly. We have noticed an adaptation phase in the first few weeks of HFD [12], which we now wish we had analyzed more closely instead of beginning another cohort of animals through the long and costly procedure. Therefore, all of the nonterminal procedures, weight gain, blood glucose, insulin, adiponectin, echocardiography, DEXA, and blood pressure (noninvasive tail cuff), should be conducted weekly. A few intervening dates should also be selected for tissue collection. Terminal assays that should be considered at these intervening dates are liver, skeletal muscle, and adipose deposits for weighing, histology, and immunoblot analysis. Interesting results would include identifying changes in molecules used by the tissues in attempts at adaptation. For example changes in enzymes and molecules controlling glucose and lipid metabolic rates would be of interest to investigate early in the HFD protocol.

A recent chapter from Baribault describes the GTT, ITT, insulin secretion tests, nonesterified free fatty acid quantification, body composition, and terminal harvesting techniques [4]. As this chapter appears in Methods in Molecular Biology and contains protocol details we will not repeat these descriptions here.

Although many of these tests appear straightforward some additional considerations are important. Animal mass can be reported as absolute or relative to CD mice or relative to beginning masses. There are pros and cons to each reporting method. Perhaps the most accurate is comparing the ratio of the animal's final weight to the animal's own beginning weight, such that the animal serves as its own control. However, using this method may not be the optimum method to compare between sexes or mouse strains where an absolute change would be optimal.

Another consideration for many of these tests is the time of day performed and if any fasting is required. As mice are nocturnal many of their diurnal cycling hormones and activities would be opposite of those found in humans. Therefore, as with all biological experiments the time of day must be kept consistent. Multiple publications discuss the appropriate length of fasting to achieve the most accurate data for various tests. Fasting is required to minimize variation and therefore minimize the number of animals needed to uncover differences. Fasting is usually used with the basal blood glucose measurements, GTT, ITT, and acute ITT. Fasting overnight is more extreme than fasting in the daylight hours. I have found the discussion given by Dr. Andrikopoulos et al. extremely helpful [51]. These authors empirically identified 6 hours of fasting and 2 g/kg of oral glucose as the most informative assay parameters. The most often used insulin dose is 1 mU/g of Humalog (E. L. Lilly, Indianapolis) for ITT [4, 12].

Another important set of characteristics can be achieved using insulin and glucose* clamp experiments*. These experiments require additional equipment and are not high-throughput. In brief, for a hyperinsulinemic euglycemic clamp, the animals receive a basal insulin delivery (3 mU/min/kg) which is a commonly used dose for rats [98] and the amount of glucose required to keep the blood sugar in a certain range is quantified. These studies quantify insulin sensitivity better because they circumvent the counterregulatory responses associated with ITT. Additionally, a hyperglycemic clamp technique can also be used. Clamp techniques are often used in conjunction with labeled glucose so that individual tissues can be analyzed for glucose uptake.

Various methods can be used to determine the* body composition* of mice. The most high-throughput method is simply weighing some of the various fat masses and comparing this to the animal's body mass, both measurements should be determined anyway as part of the harvesting protocol. The next upgrade is to utilize DEXA measurements on all or a consistent portion of the mouse. This technique has the added bonus that it is not a terminal procedure and a time course can be constructed for each animal. The gold standard for body composition is an MRI. This also benefits from being amenable to establish a time course, although it is the furthest from high-throughput of the three methods and may also be expensive.

## 5. Tissues Affected by HFD

Human type 2 diabetes mellitus is truly a systemic, multiorgan disease and each of these organs is also negatively affected in mouse models on a HFD. The mouse organs affected each are being investigated to gain pathologic and therapeutic knowledge into the disease. The ultimate goal is to identify therapeutics which reduce all systemic and organ pathologies. In this section additional, tissue-specific analysis methods will also be listed and referenced or discussed.

### 5.1. Adipose Tissues

Adipose tissue pathology is at the forefront of HFD investigations and the subsequent pathologies. Extensive data identifies that adipose inflammation, including secretion of adipokines and activation of adipose resident macrophages, is pathogenic for all other tissues and organs [99]. The different types of adipose tissues should be considered separately [62]. It is indicated that visceral white fat is the most pathogenic type because it secretes large amounts of adipocytokines such as leptin, TNF*α*, IL6, and adiponectin that can disrupt homeostasis when dysregulated due to HFD [45]. As the visceral fat undergoes hypertrophy and perhaps hyperplasia it secretes more of these potentially unhealthy adipocytokines. Subcutaneous adipose tissue also secretes the adipocytokines but in smaller quantities so this adipose site is not as frequently studied. The adipocytokines are reliably measured by serum or tissue extract enzyme-linked immunosorbent assays (ELISAS). The weights of the various adipose deposits are also very important to obtain at harvest time.

Interesting work is also coming forward from fat deposit transplantation studies. Relatively small amounts (0.1 mg) of brown adipose tissue were transplanted into the visceral cavity of HFD recipients. Eight weeks after surgery the recipients had significant improvements in glucose tolerance and at 12 weeks the recipients had significant improvements in insulin tolerance tests [100]. A detailed review of brown adipose tissue physiology assessment has recently been published [101] and will therefore not be repeated here.

Insulin resistance in adipose tissues is pathogenic for the remainder of the organism. Adipocytes usually respond to insulin by dividing, increasing glucose uptake, and inhibiting lipolysis [45]. When the cells become insulin resistant due to HFD the body is subjected to increased levels of hyperglycemia and lipotoxicity because of the excessive lipolysis.

### 5.2. Skeletal Muscle

As skeletal muscle is the primary tissue for glucose disposal, its insulin resistance is systemically pathogenic. Skeletal muscle also provides one of the best methods to cure T2DM through increasing exercise and disposing of glucose in an insulin independent manner, thus by passing the pathology and reestablishing homeostasis. For these reasons investigating and treating the skeletal muscles of HFD mice are critically important.

Skeletal muscle of HFD mice succumbs to many of the above-mentioned pathologies: insulin resistance, lipotoxicity from excessive lipid storage, and inflammation. Insulin resistance can be measured with an acute ITT, fifteen minutes after an insulin bolus (1 mU/g); skeletal muscle is harvested and immunoblots are used to determine the levels of phosphorylated pAkt at T^308^ and S^473^ [45]. Insulin resistant tissues produce less pAkt and this appears to be a linear relationship so that the degrees of insulin resistance can be quantified [45]. Other downstream targets of the insulin receptors can also be monitored for insulin signaling, although specificity of insulin signaling must be considered during data analysis [45]. Increased lipid levels can be analyzed by a number of methods. Histologically the Oil Red O stains can be used to quantify cellular lipid droplets. The gold standard is perhaps by electron microscopy so that lipid droplet location can also be determined. Lipid droplets in the mitochondria [12, 16] versus cytoplasmically located [102] would reveal specific pathologic mechanisms. Inflammation and specific cells of the invading immune system can be measured by histology or flow cytometry.

### 5.3. Liver

The liver of HFD mice is negatively affected in a number of ways [21, 103]. As with many HFD-elicited diseases the liver pathology is progressive. The pathology usually begins with patchy steatosis/fatty droplet accumulation (nonalcoholic fatty liver disease (NAFLD)), inflammation (pathogenic cytokines and ROS release), fibrosis, hepatocyte hypertrophy and hyperplasia, more global steatosis (nonalcoholic steatohepatitis (NASH)), liver mass gain, cirrhosis, and finally hepatocellular carcinoma (HCC). The usefulness of a long-term HFD to model the steps of this disease progression is discussed in a review by Kanuri and Bergheim [103].

The HFD liver is also systemically pathogenic. Insulin usually reduces hepatic gluconeogenesis and glycogenolysis. Therefore, in an insulin resistant animal or T2DM patient the liver actually makes and secretes more glucose than normal [104]. In an organism that is already hyperglycemic the liver adds even more sugar to the blood stream. Insulin signaling in the liver also increases lipogenesis. However, due to “selective insulin resistance” this signaling pathway is not inhibited during times of systemic insulin resistance and therefore the liver inappropriately produces lipids in response to the hyperinsulinemia in the later stages of disease [105]. In addition, a large proportion of a health body's readily available lipid is stored in the liver; the liver is therefore highly susceptible to lipotoxicity.

Of interest is that full liver pathology—HCC—requires a long HFD protocol to be established; at 60 weeks of HFD still only 54% of the mice displayed HCC [21]. In addition, liver cirrhosis in mice is rarely detected [21]. However, this HFD model is still very useful in liver pathology and treatment investigations. For example, long-term (60 weeks) HFD feeding establishes NAFLD, including the HCC, in just over half of the HFD C57Bl/6 mice [21]. The group went on to use this HFD mouse model, at multiple HFD durations, to demonstrate the preclinical efficacy of metformin treatment in reducing HCC if the treatment starts coincident with the diet, but not if metformin treatment is begun after NAFLD has begun to develop [57]. These authors also compared various time points of diet duration and treatment to elucidate the mechanisms behind metformin cancer reduction.

The progression of different liver pathologies can be evaluated in a number of ways. Histology is very useful to identify the stage of disease progression and to quantify the degree of pathology. Histology can identify amount of excessive lipid storage, steatosis, and HCC. The liver is also well studied by the acute ITT to quantify its level of insulin resistance.

### 5.4. Cardiac

As many T2DM patients die of cardiomyopathy without vessel disease or hypertension, investigating diabetic cardiomyopathies is of high importance [106]. Multiple studies have investigated the effects of HFD upon cardiac function, remodeling, and metabolism. Mice fed a HFD for 10 weeks develop myocardial insulin resistance evidenced by a downregulation of insulin receptor activity, downregulation of AKT signaling, and increased fatty acid oxidation [107].

Lipid accumulation in the heart occurs when lipid intake exceeds lipid metabolism. It is exacerbated in chronic (HFD) situations and obesity when the body is overwhelmed by free fatty acids (FFA). Even though the heart metabolizes lipids for 60–80% of its total basal energy it will store excess FFA and metabolites. Abnormal accumulation of nonoxidative lipid derivatives leads to increased apoptotic signaling, oxidative stress, and broad cellular dysfunction in the heart [108]. Overwhelming evidence indicates that the type of ingested fat is critically important for its place in the pathogenic or protective spectrum (reviewed in [69]). This is especially evident in the effects of particular fats in the diets on cardiac function. For example, a diet high in saturated fats is protective in muscular dystrophy mediated cardiomyopathy in hamsters [109].

Rodent HFD models of T2DM also reproduce the “obesity paradox,” initially identified in humans [110]. The situation is labeled a paradox because obese patients are better at overcoming long-term damage after myocardial infarctions. In rats subjected to pressure-overload hypertrophy, those receiving HFD are protected from cardiac hypertrophy and dysfunction [69]. Furthermore, mice on a HFD have less postinfarct myocardial remodeling and dysfunction compared to normal diet controls [111]. The similarities between the human diseases and the rodent models facilitate investigations into the mechanisms of this paradox and identify if it can be leveraged for human therapeutics.

Multiple investigations failed to identify cardiac pathologies after long-term HFD feeding. For example, in a 6-month HFD study the investigators did not find any cardiac pathology by echocardiography [19]. Alternatively, other investigators have identified pathologies within shorter HFD protocols. For example, in a 12-week HFD study we identified cardiomyocyte hypertrophy, unsuccessful metabolic adaptations, and lipid droplet accumulation [16]. These results indicate that caution must be used when designing HFD experiments, especially for diabetic cardiomyopathy assessments. It may be that in these long-term experiments the hearts have compensated fully and have established a new homeostasis and the pathology must be evaluated over a time course. This is an interesting point requiring further study, because such compensatory mechanisms may reveal therapeutic targets. A very interesting study was conducted by Calligaris et al. These authors compared 8, 12, and 16 months of HFD on the C57Bl/6 male hearts. They identified cardiac remodeling at all time points, which became more severe as the diet duration was extended. In the HFD group they identified functional pathology, but only after dobutamine pharmacological stress [18].

There are a number of possible reasons for the discrepancies regarding HFD-induced DCM. Some of the reasons are easily identified: different diets (fat type is highly important, W. C. Stanley), diet duration, different assays, and age of animals. Some of the harder to identify reasons are genetic drift, seasonal variation, and diurnal variation in time of assays. When designing a HFD protocol these variations each must be considered and minimized as much as possible.

The heart must be further evaluated by a number of unique methods [5].* Cardiac pathology assessments* are critically important to obtain due to the large number of T2DM patients dying from heart failure [106]. Echocardiography (Echo) is an essential assessment method for T2DM. Echo indicates morphometric hypertrophy and dilation and provides information on cardiac function. DCM is defined as reduced cardiac function without hypertension or vessel disease. Therefore, blood pressure is also a key assessment for DCM. Generally, hypertension is not identified in HFD mouse models [16]. However, this needs to be assessed for each experiment. Blood pressure values can be quantified by either terminal (pressure volume loops (PV loops)) or survival (tail cuff) techniques. The tail cuff procedure benefits from being survival, relatively high-throughput, and inexpensive while the PV loops benefit from being more accurate and accepted by reviewers. The best possible scenario would be to perform tail cuff weekly throughout the HFD protocol and perform the terminal PV loops at the termination of the procedure.

Hypertrophy is a common sequela of HFD. Hypertrophy can occur in two distinct or overlapping characteristics at the organ level and/or cellular level. The organ level hypertrophy can be assessed with Echo and heart weight to tibia length and the cellular hypertrophy can be assessed by histology methods and ImageJ [16].

### 5.5. Pancreas

The pancreas is also affected by the HFD. The hyperglycemia caused by the HFD signals the pancreas to secrete more and more insulin. Eventually the pancreas falters and hyperinsulinemia is followed by hypoinsulinemia. These changes in insulin levels are seen in patients and in mice fed a HFD.

Of course, because T2DM is a systemic disease, all cells and tissues are affected by the disease-defining hyperglycemia and lipotoxicity [45]. Principal among these cell types would be endothelial cells, neurons, and beta cells of the pancreas.

## 6. Conclusions and Future Directions

The HFD mice have proven invaluable not only for the identification of molecular pathways affected by HFD, but also in preclinical protocols to test potential therapies to reverse the condition and its many associated pathologies [4]. As the research community identifies the most human-like HFD model the strength of using this model for preclinical protocols can only become better.

Many therapeutics are being tested in the HFD protocol. Recently resveratrol (RSV, red grape extract) has come into the news as being antiaging and potentially inhibiting T2DM symptoms. When given for the 12 weeks of HFD, RSV produced only a slight reduction of body weight and blood glucose but had a significant reduction on renal fibrosis and renal dysfunction [112]. Through further experiments, the authors attributed the benefits to inflammation reduction. Other pharmacologies that have been tested in the HFD protocol include metformin, ezetimibe, acarbose, and atorvastatin.

As the research community approaches the ideal HFD protocol, consistent protocols will be undeniably helpful. As stated previously this would enable ease when comparing study to study, especially important for the preclinical therapeutic trials. I will now go out on a limb and steel myself for the upcoming onslaught of detractors. I propose this following standard protocol:

- Using C57Bl/6J.
- Feeding from 4 to 20 weeks old.
- Using both male and female mice.
- Using a HFD plus high fructose to mimic the human diet best.

This protocol can be used to compare the many therapeutics. It can be also modified to study specific mouse strains, exercise, age, length of diet, and so forth. For example, it would be of great interest to compare young and old mice in a HFD protocol. Although older mice do not have the high levels of chronic inflammation as found in the aged human population, it would be of interest to establish if the older mice develop T2DM symptoms at a shorter HFD time period.

## Figures and Tables

**Table 1:** Considerations for designing a high fat diet protocol.

	Notes	References^#^
Design		
Length of diet	Adaptive (<1 week)	
	Acute (<2 weeks)	
	Chronic (>8 weeks)	
Formulation	Matching Human Western Diet	
	Accentuate pathology	
Age	Pathologies intensify with age	
Gender	Males are predominantly used to avoid the female cycles	
Strain	Hyperglycemia susceptible: C57Bl/6J (C57Bl/6N healthier than C57Bl/6J after HFD), DBA2/J, BXD66	[12, 13, 48–50]
	Hyperglycemia resistant: MRL, Lrg, A/J, BXD77, BALB/cJ	

Assessments		
Weights	Whole body and specific tissues: different adipocyte sites, liver, heart	
Weekly blood glucose	Fasting versus fed, time of day	
Echocardiography		
Blood pressure	PV loops versus noninvasive	
Glucose TT	Fasting versus fed, time of day, acute versus long term	[44, 51]
	Glucose delivery: oral, IP, or IV	
Insulin TT	Fasting versus fed, time of day, acute versus long term	
Serum collection	Fasting versus fed, time of day	
DEXA	Allows time course of body composition	
NMR	Allows time course of body composition, more reliable than DEXA	[44]
Histology	Fibrosis, picrosirius red, periodic acid shift, immune infiltrate, adipocyte, skeletal muscle, cardiac cell size	
Fibrosis	TGF*β*, pSMAD2/3, *α*SMA, IL6, IL13, IL33	[48]

Additional phenotypes		
Hyperglycemic clamp	Identifies glucose sensitivity, by measuring serum insulin levels	[44]
Hyperinsulinemic-euglycemic clamp	Considerations are time of clamp and how tightly to regulate the glucose infusions	[44, 52, 53]
	Determines glucose use	
Tissue specific glucose uptake	Used in conjunction with the above clamp techniques	[44]
	Requires an MRI and radioactively labeled energy sources	
	Much easier in rats	
	Determines insulin sensitivity in multiple tissues	
Inflammation	Flow cytometry for specific cell types	[48]
	Histology can also identify specific cells	
	Cytokine levels in serum or tissue extracts	
Liver, NAFLD	Aspartate transaminase and alanine transaminase	
	Progression of disease: steatosis > steatohepatitis > cirrhosis > HCC	
Electron micrographs	Analyze mitochondria and lipid storage sites in detail	

TT, tolerance test; DEXA, duel-energy X-ray absorptiometry; HCC, hepatocellular carcinoma; IP, intraperitoneal; IV, intravenous; NAFLD, nonalcoholic fatty liver disease. ^**#**^References are given where inappropriate.

**Table 2:** Common diet durations.

Duration	Key findings	References^#^
12 weeks	Identified diabetic symptoms in wild type C57BL/6 mice: these were not found in the wild type MRL mice.	[12]
	Identified diabetic cardiomyopathy symptoms in wild type C57BL/6 mice: these were not found in the wild type MRL cardiac tissue.	[16]
	Identified sulforaphane (an antioxidant) decreases diabetic cardiomyopathy in a HFD plus streptozotocin model.	[54]

16 weeks	In WT C57BL/6 HFD increased LVW by 21%. In WT C57BL/6 HFD increased liver weight by 86%.	[55]

12-, 16-, and 20-week comparisons	Epididymal AT death peaks at 16 weeks of HFD, compared to 12 and 20 weeks. This is coincident with a peak of AT macrophages. Inguinal AT death was less pronounced at all times tested.	[56]

24 weeks	Identified that a Th1 immune response caused mice to be more susceptible to HFD pathologies.	[48]

8-, 12-, and 16-month comparisons	At 8 months hyperglycemia, hyperinsulinemia, and hypercholesterolemia and insulin resistance were found. Cardiac remodeling by echo was also identified. At 16 months the authors also report cardiac metabolic compensations and tissue remodeling in the form of fibrosis and hypertrophy.	[18]

8-, 30-, and 60-week comparisons	NAFLD and HCC established at 30 weeks and significant liver pathology observed at 8 weeks of HFD.	[21, 57]

6 and 16 weeks	Identified liver pathology only at 16 weeks and only in males.	[58]

6 weeks	Increased LV mass and reduced FS.	[59]

AT, adipose tissue; FS, fractional shortening; HCC, hepatocellular carcinoma; LV, left ventricle; NAFLD, nonalcoholic fatty liver disease. ^**#**^References are given where inappropriate.

## Abbreviations

AITT: Acute insulin tolerance test
AGTT: Acute glucose tolerance test
B6: The C57Bl/6J mouse strain from Jackson Labs
DCM: Diabetic cardiomyopathy
DM: Diabetic mellitus
HCC: Hepatocellular carcinoma
HFD: High fat diet
GTT: Glucose tolerance test
ITT: Insulin tolerance test
NAFLD: Nonalcoholic fatty liver disease
RSV: Resveratrol
T2DM: Type 2 diabetes mellitus.

## Additional Points

This is a guide to plan your high fat diet mouse experiments.
